# Drop dead! Female mate avoidance in an explosively breeding frog

**DOI:** 10.1098/rsos.230742

**Published:** 2023-10-11

**Authors:** Carolin Dittrich, Mark-Oliver Rödel

**Affiliations:** ^1^ Museum für Naturkunde Berlin, Leibniz Institute for Evolution and Biodiversity Science, Invalidenstraße 43, 10115 Berlin, Deutschland; ^2^ Department of Biology and Environmental Sciences, University of Jyväskylä, Jyväskylä, Finland; ^3^ Berlin–Brandenburg Institute of Advanced Biodiversity Research (BBIB), Berlin, Germany

**Keywords:** death feigning, European common frog, experience, sexual conflict, thanatosis, tonic immobility

## Abstract

Males’ and females’ reproductive strategies may differ, potentially leading to sexual conflict. Increased efforts by males (harassment, forced copulation, intimidation) to gain access to females could even negatively affect female survival and thus lead to reproductive failure for both individuals. In anurans, a higher mortality risk of mating females has been reported in explosive breeding species. During these mating events, several males cling to a female, which are mostly unable to get rid of the unwanted males. This can lead to the female's death. From the literature, it seems that females of explosive breeding frogs have no means to reject unwanted males. Here we describe female mate avoidance behaviours in the European common frog. We observed three female avoidance behaviours, namely ‘rotation’, ‘release call(s)’ and tonic immobility (death feigning). These behaviours were significantly associated with smaller female body size, and smaller females were more successful in escaping amplexus. Tonic immobility as a tactic to avoid mating or male harassment has only been observed in a handful of species and only in one other amphibian. Our observations show that females in explosive breeding frogs may not be as passive and helpless as previously thought.

## 1. Background

In classical sexual selection theory, sexual selection is mostly driven by female choice and male combat [1]. According to this theory, both sexes aim to maximize their reproductive fitness. Whereas males could mate with several females to increase the number of offspring, females are limited by their reproductive cycle and the number of potential offspring within one of those cycles, which is usually unchangeable [2]. Hence the costs, benefits and interests of both sexes may differ, which could lead to sexual conflict [3] and sexual coercion [4]. The most dramatic examples of sexual conflict are infanticide, i.e. the so-called ‘Bruce effect’, where the death of offspring stimulates ovulation in the mothers of some mammalian species [5,6], or the forced copulation behaviour of male waterfowl, which leads to an arms race reducing the risk of forced copulation and unwanted fertilization [7]. Thus, sexual conflict directly generates selective pressures and affects evolutionary processes [8]. Increased efforts by males (harassment, forced copulation, intimidation) to gain access to females could lead to reproductive failure for individuals involved and even negatively affect female survival (ducks: [9], frogs and toads: [10], mammals: [11]).

In frogs and toads, a higher risk of mortality in mating females has been reported, particularly in explosive breeding species. This breeding system is characterized by an (often) male-biased operational sex ratio, synchronized female receptivity and low sexual selection [12]. In explosive breeding anuran species, pair formation is mainly driven by male scramble competition for females. Females could either be attracted by male advertisement calls or remain passive, depending on the density at the breeding site [13]. Being active as a female includes the risk of being amplected by several males [13], which could lead to death of the respective female [10,14,15].

The European common frog, *Rana temporaria* Linnaeus, 1758, is an explosive breeder, forming dense breeding aggregations in early spring [16]. Recent literature, including comprehensive reviews, states that females are passive during courtship and reproductive behaviour [16], which is attributed to sexual coercion, particularly female harassment, by males. However, there is published evidence that *R. temporaria* females are not passive during reproduction. More than 250 years ago, Rosenhof [17] reported that females produce grunting sounds like male release calls [18]. This behaviour was observed after females have deposited eggs, thus signalling non-receptivity to a male [19]. In addition to these calls, Savage [19] observed females showing tonic immobility (we herein use this neutral term instead of the more anthropocentric ‘death feigning’) [20], when grabbed by a male, sometimes for several hours. Female common frogs are therefore unlikely to be defenceless against sexual coercion by males.

Here we describe female mate avoidance behaviours observed during a recent study of male mate choice [21]. Our observations suggest that female common frogs are not as passive as is usually assumed, and that age and experience may play a key role in the performance of these behaviours.

## 2. Methods

In spring 2019, we collected male and female common frogs (*Rana temporaria*) to test whether males show a preference for female body size [21]. We placed two differently sized females together with a male in a water-filled box (40 × 60 × 40 cm, 5 cm of water) and allowed them to move freely for one hour. We recorded the mating behaviour in each box using a webcam (LogiTech 920). Female avoidance of male mating attempts was observed by reviewing the video footage. For details on location and handling see Dittrich *et al*. [21]. All raw data and scripts for reproducing the analysis are stored in an online repository [22].

We counted the occurrence of behaviours alone, and in all possible combinations (e.g. calls with rotation and/or tonic immobility, etc.). We used a pairwise t-test with p-adjustment for multiple testing (false discovery rate) [23] to determine whether female body size differed when performing specific behaviours. We tested this for the most common types of behaviours (sample size greater than five). We modelled escape probabilities using a binomial logit model for female body size *per se* and the body size ratio between females and males. A body size ratio above one means that the female is larger than the male, and vice versa if below one. We used the R statistical environment [24] for all analyses and graphs. We used the packages ggplot2 [25], gridExtra [26], png [27] and ggsignif [28] to plot graphs, MuMIn [29] to calculate the likelihood-ratio adjusted *R*^2^ of the binomial models and plyr to count the number of occurrences [30].

## 3. Results

We observed three female avoidance behaviours, namely ‘rotating’, ‘release call(s)’ and tonic immobility. We defined ‘rotating’ as a female starting to rotate around her own body axis when amplexed by a male, while the male tries counteracting the rotation with its hind feet (electronic supplementary material, video S1). Release calls are emitted when the female is amplexed by the male and she begins to call. Two different calls are produced, a grunt and a squeak (electronic supplementary material, audio S2 and S3, see details in [18]). Tonic immobility is defined as the stiffening of the female—arms and legs outstretched from the body—after being amplexed by a male (electronic supplementary material, video S4). All three behaviours were observed when the frogs were in water, but we also observed one instance of tonic immobility in the terrestrial habitat, between an 83 mm male and a 79 mm sized female (electronic supplementary material, figure S5).

**Figure 1.**
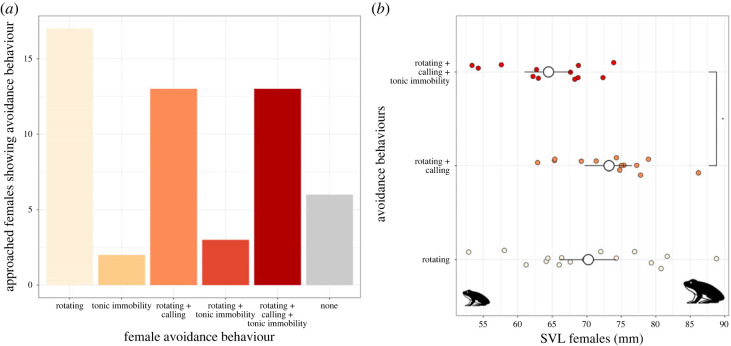
(*a*) Count data for all different avoidance behaviours in common frog females, (*b*) female body size in mm and avoidance behaviour displayed. Females displaying all three behaviours were on average significantly smaller than females displaying rotating and calling (pairwise *t*-test with fdr correction, *p* = 0.02). Large white dots represent mean body size, whiskers the 95% confidence interval. Dots are jittered for better visibility.

In our experiments, a total of 54 females were amplexed by a male. The most common female mate avoidance behaviour was rotating, which was exhibited by 83% of all amplexed females, either alone or in combination with the other two behaviours. We also observed female body rotation in the natural habitat several times, suggesting that it is a common behaviour. Release calls were emitted by 48% of amplexed females, and calls were always associated with rotating (figure 1*a*). Tonic immobility occurred in 33% of all clasped females, most often in combination with rotating and calling (13/18). The occurrence of all three behaviours together was significantly associated with smaller female body size, whereas rotating in combination with calling was associated with larger female body size (pairwise t-test with fdr correction, *p* = 0.02, figure 1*b*).

**Figure 2.**
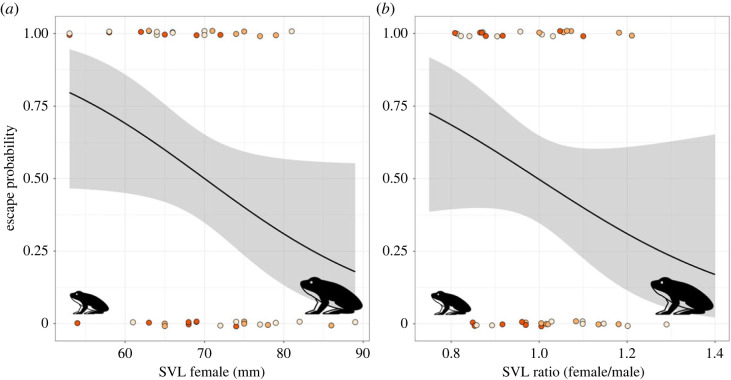
(*a*) Escape probability of female common frogs during avoidance behaviour as a function of female body size (SVL), or (*b*) the female-to-male SVL ratio. Dots represent individual females and their respective avoidance behaviour, light orange = rotating, orange = rotating and calling, red = rotating, calling and tonic immobility. Dots are jittered for better visibility.

Displaying of mate avoidance behaviour resulted in the escape of 25 females (46% of all females in amplexus). The probability of escape showed a trend to increase with decreasing female body size (binomial GLM; *p* = 0.06, figure 2*a*), and with decreasing female-to-male SVL ratio (smaller females, binomial GLM; *p* = 0.09, figure 2*b*).

## 4. Discussion

European common frog females displayed body rotations, release calls and tonic immobility in order to escape amplecting males. Small females used all three mate avoidance behaviours more frequently than larger females and had higher escape probabilities.

While several mate avoidance behaviours were exhibited by females, body rotation was the most common behaviour when a female was clasped by a male. In order to maintain an upright position and to keep holding the female, males counteracted the rotation by using their stretched hind feet. This behaviour has been described by Savage [19] but was never subsequently reported or cited. There could be several reasons for the effectiveness and high frequency of female rotation. Rotating the male under the female and thereby under water might force males to release females to avoid drowning and enables females to breathe. Female rotations could also be a strategy testing the strength and endurance of males. Mating with a stronger male could increase a female's survival probabilities. If a male can kick-off rivals and thus prevent the formation of a ‘mating ball’, the female is more likely to survive the reproductive season [10,14,15]. Females may therefore test potential mates. However, testing potential mates can also be costly [31], particularly if a female receives more attention through this behaviour but cannot defend herself against sexual coercion from multiple males. It remains to be determined which (phenotypic or genotypic) trait(s) is (are) under selection, leading to mate acceptance or rejection, and what benefits and costs, if any, are associated with mate choice by female *R. temporaria*. There seem to be no obvious direct benefits of choosing a particular male, as males provide no parental care or defend any resources. Direct benefits, such as increased fertilization success by larger or size-assorted males have been disproved; in fact, fertilization success has been found to be independent of size assortment [32]. We also detected a high rate of multiple paternity in our study population (80% [33]), probably due to ‘stray sperm’, which would minimize the potential adaptive effects of precopulatory female mate choice.

Interestingly, smaller female common frogs were more successful at escaping an amplexus by rotating than larger females. This may be due to mechanical grip characteristics. If females are (much) smaller than the amplexing male, the male may not be able to hold them tight enough to maintain the amplexus. In Cane toads (*Rhinella marina*) it has been shown that males with shorter arms are better at holding onto females than males with longer arms. The latter are more often replaced by other males because they cannot hold the females properly [34]. Likewise in the Common toad (*Bufo bufo*), male takeovers were more successful when pairs were not size assorted, i.e. size mismatch led to weaker bonding in amplexus [35]. In our study population, we found size-assortative mating [21,32], where the males are usually smaller than the females. A higher escape probability for smaller females amplexed by larger males could be due to mechanical grip properties of the pair and supports the previously documented size-assortative patterns in the absence of male mate choice [21,32].

Tonic immobility is known to occur throughout the animal kingdom, ranging from invertebrates to vertebrates [20]. It is mainly interpreted as a defensive strategy against predation [36]. As a stress response to an immediate threat of predation (or a strong tactile stimulus), it is an ‘evolutionary conserved defensive mechanism of last resort’ [37]. Tonic immobility as a tactic to avoid mating, reproductive cannibalism or male harassment, has been observed in a handful of species, mainly in arthropods such as spiders [38] and dragonflies [39], and in one other amphibian species, the sharp-ribbed newt (*Pleurodeles waltl*; [40]). Savage [41] reported a female *R. temporaria* that showed tonic immobility (feigned death) for two hours after being amplexed by a male. The male called frequently during this time but did not let her go. We have observed tonic immobility by females frequently, but more often in smaller and therefore younger females. The smaller females also showed the full repertoire of behaviours more often than the larger females. We therefore speculate that this behaviour may be related to age or experience of females. Stress increases corticosterone circulating in the blood, which could inhibit reproductive behaviour [42] and is positively correlated with the duration of tonic immobility [37]. Less experience with reproduction may result in more stressed females [20,43,44]. In the American wood frog (*Lithobates sylvaticus*), a species which is very similar in morphology and behaviour to the European common frog, females showed a higher stress response during breeding when male scramble competition was high [45]. An important source of stress, that could trigger tonic immobility, is the frequently observed formation of ‘mating balls’, which often result in the death of females and males by drowning [10,14,15]. Whether drowning (lack of oxygen) is the main factor leading to death is not entirely clear, especially as this species is able to absorb oxygen from the water through the skin and partly hibernates in ponds or streams [41]. It may be a mixture of factors that lead to the death of individuals during these mating balls. Tonic immobility can lead to bradycardia and therefore an increase in peripheral resistance [37], which could reduce oxygen uptake through the skin. During the fight, water may enter the lungs, further reducing oxygen uptake. Breeding begins immediately after hibernation and individuals do not feed before joining the breeding group [46], resulting in high energy demands that may not be met, leading to allostatic overload and death [47]. Another factor could be the high pressure exerted by the amplexed males, which could damage the female's organs and lead to egg extrusion [10]. Anyhow, what the proximate factor killing the females may be, tonic immobility may be a better option for a female than fighting her way out of the amplexus, as any movement in a large mating group automatically attracts attention of further nearby males and thus increases the probability of a mating ball formation. With these considerations in mind, it would be interesting to measure stress corticosterone levels in female *R. temporaria* and investigate how they correspond to age and sex ratio in breeding aggregations [45,48].

Future studies should consider investigating different frog densities, sex ratios and age classes, to test for mate choice and the potential costs associated with reproduction. However, our study provides clear evidence that female frogs, even in dense mating aggregations of explosive breeders, are less helpless than generally assumed.

## Data Availability

The raw data are deposited together with a readme text file of metadata, description of variables and the R-script to reproduce all analysis and figures. The DOI is provided in the references [22]. https://doi.org/10.7479/s00f-ta45. The audio and video files are provided in electronic supplementary material [49].
